# A novel minimally invasive and versatile kyphoplasty balloon-based model of porcine spinal cord injury

**DOI:** 10.3389/fneur.2024.1422357

**Published:** 2024-07-16

**Authors:** Sean M. Barber, Tatiana Wolfe, Alexander G. Steele, Kris Hoffman, Matthew K. Hogan, Allison Frazier, Xiufeng Tang, Dimitry G. Sayenko, Philip J. Horner

**Affiliations:** ^1^Department of Neurosurgery, Houston Methodist Neurological Institute, Houston, TX, United States; ^2^Center for Neuroregeneration, Houston Methodist Research Institute, Houston, TX, United States; ^3^University of Arkansas for Medical Sciences, Psychiatric Research Institute, Brain Imaging Research Center, Little Rock, AR, United States

**Keywords:** spinal cord injury, animal model, minimally invasive surgery, kyphoplasty, spinal cord contusion injury

## Abstract

**Introduction:**

Spinal cord injury (SCI) animal models often utilize an open surgical laminectomy, which results in animal morbidity and also leads to changes in spinal canal diameter, spinal cord perfusion, cerebrospinal fluid flow dynamics, and spinal stability which may confound SCI research. Moreover, the use of open surgical laminectomy for injury creation lacks realism when considering human SCI scenarios.

**Methods:**

We developed a novel, image-guided, minimally invasive, large animal model of SCI which utilizes a kyphoplasty balloon inserted into the epidural space via an interlaminar approach without the need for open surgery.

**Results:**

The model was validated in 5 Yucatán pigs with imaging, neurofunctional, histologic, and electrophysiologic findings consistent with a mild compression injury.

**Discussion:**

Few large animal models exist that have the potential to reproduce the mechanisms of spinal cord injury (SCI) commonly seen in humans, which in turn limits the relevance and applicability of SCI translational research. SCI research relies heavily on animal models, which typically involve an open surgical, dorsal laminectomy which is inherently invasive and may have untoward consequences on animal morbidity and spinal physiology that limit translational impact. We developed a minimally invasive, large animal model of spinal cord injury which utilizes a kyphoplasty balloon inserted percutaneously into the spinal epidural space. Balloon inflation results in a targeted, compressive spinal cord injury with histological and electrophysiological features directly relevant to human spinal cord injury cases without the need for invasive surgery. Balloon inflation pressure, length of time that balloon remains inflated, and speed of inflation may be modified to achieve variations in injury severity and subtype.

## Introduction

The annual incidence of spinal cord injury (SCI) in the United States is estimated at 54 cases per one million people, totaling around 18,000 new cases annually (1). SCI can result in enduring sensory and motor alterations, causing partial or complete paralysis, sensory loss, autonomic dysreflexia, bowel and bladder changes, and pain (2). These impairments significantly impact an individual’s quality of life, necessitating continuous rehabilitation efforts to maintain the ability to perform daily tasks. The occurrence and severity of post-injury impairments vary based on the level and extent of the injury. The precise pathophysiological mechanisms at work in SCI and their relationship to the clinical phenotype of a patient with SCI are currently underexplored. Despite a clinical diagnosis of “complete loss” of sensorimotor function in some patients with SCI, evidence suggests that many sub-functional neural connections between the brain and spinal cord remain intact across the injury site (3–5). These connections are not robust enough to drive clinically detectable function; however, they are capable of influencing the excitability of spinal sensorimotor networks below the lesion (6–8). Crucially, the decrease of descending pathways’ integrity and cortical grey matter volume is directly correlated with spinal cord atrophy, suggesting that trauma-induced spinal degenerative processes spread towards the brain. These changes appear to be dynamic and influenced by the level, completeness, and time after injury, as well as the extent of clinical recovery (9). These findings are critically relevant to our study, as they indicate that (1) brain networks involved in different demands of motor control remain responsive even in chronic paralysis, (2) therapeutic strategies aimed at restoring spinal cord function, even in individuals with chronic SCI, can build on preserved competent descending control, and (3) the heterogeneity in residual motor function associated with SCI and individual response to a therapy can be addressed using mechanistic neurophysiology studies.

Research into the pathophysiology of SCI and therapeutics to spur regeneration of the injured spinal cord has drawn in large part from animal studies. The development of animal models for SCI that reliably and accurately reproduce the sequelae of SCI in humans is thus crucial to our progress towards effective treatments. The large majority of contemporary animal models for SCI rely on an invasive surgical procedure (i.e., laminectomy) for access to - and reproducible mechanical deformation of - spinal tissue (10). While laminectomy is useful in that it allows direct visualization and manipulation of the spinal cord, the procedure itself also imparts some degree of animal morbidity, may impact animal recovery/functional status, and demonstrates limited clinical correlation to the closed, compressive SCIs commonly seen in humans. Furthermore, though the effect of laminectomy itself on the injury and recovery pattern itself after SCI has not been well studied, laminectomy is often indicated as a surgical treatment in patients with spinal cord injury who are found to have persistent spinal cord compression, and as such, laminectomy could conceivably have an effect on functional recovery after injury creation. Additionally, laminectomy may lead to changes in spinal cord perfusion, cerebrospinal fluid (CSF) flow dynamics, and spinal stability that may confound the results of a study evaluating novel therapeutics or interventions even in the absence of ongoing spinal cord compression (11–14).

A variety of minimally invasive animal models for spinal cord injury have been developed, including models wherein a small laminectomy or hemilaminectomy is utilized to minimize bony removal (15, 16), and models involving microscopic dissection and/or operating techniques intended to minimize collateral tissue trauma (17). Each of these, however, still requires muscle dissection and removal of bone over the site of injury. Other models have utilized a balloon compression device inserted into the epidural or subdural space via a small hemilaminectomy performed caudal to the site of injury (18–20), which may mitigate the effect of laminectomy on CSF and vascular dynamics at the site of injury, but the methods utilized in these models nevertheless require muscular dissection, bony removal, and other collateral tissue damage that impact animal recovery and well-being in the post-injury phase.

We aimed to develop a minimally invasive large animal model of thoracic spinal cord injury that requires no muscle dissection or bony removal and can be performed safely and reproducibly in a large animal. The primary incentives for development of this new model were to minimize procedure-related animal morbidity, alleviate the confounding influence of bony removal on injury-site dynamics and maximize animal recovery after injury surgery. Subsequently, we conducted a thorough investigation into the pathophysiology of this injury model, employing comprehensive assessments such as imaging, neurophysiology, neurofunctional and histology results in five representative pigs.

## Results

Four of the five animals tolerated the procedure well. One animal expired on the day of injury while awakening from anesthesia. The precise cause of death was unclear, but was theorized to be related to spinal shock resulting from a relatively severe spinal cord injury. The animal was the youngest of the pigs included (3 months and 21 days at surgery, weighing 18 kg) and developed respiratory failure 2 h after extubation while in post-operative recovery, expiring as a result. Post-mortem examination revealed a relatively severe spinal cord injury with associated intradural and intramedullary hemorrhage, but no other systemic abnormalities to explain the respiratory failure and subsequent death.

### MRI

Immediate post-injury 1.5 T MRI demonstrated a T2-hyperintense focus within the spinal cord parenchyma at the site of injury in each animal consistent with a mild contusion injury. Endpoint T1w, T2w, T2-FLAIR, and myelin SHIFT MRI in all animals demonstrated signal changes within the spinal cord consistent with a mild contusion injury and a trend towards increase myelin SHIFT T2 signal, consistent with mildly injured myelin.

### Electrophysiology

**Figure 1:**
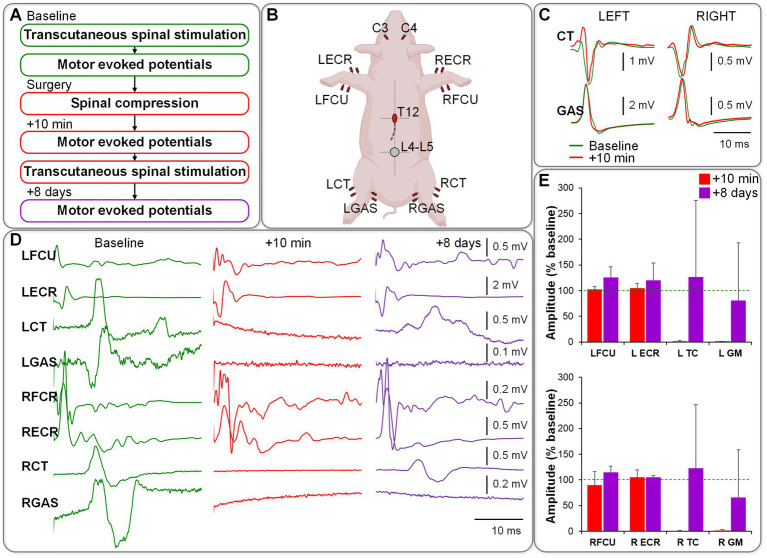
**(A)** Electrophysiology protocol/order. **(B)** Setup of stimulating and recording electrodes. **(C)** Responses elicited in hindlimb muscles during transcutaneous spinal stimulation at L4-L5 (no changes observed between baseline and post-compression). **(D)** Motor evoked responses induced during transcranial stimulation over the motor cortex (responses in hindlimb muscles were abolished immediately post-compression and partially restored on day 8 post-surgery). **(E)** Group data from *n* = 4, normalized to the baseline.

After the balloon implantation but before inflation, spinally evoked potentials were evident in recorded hindlimb muscles. Transcranial electrical stimulation (TES) induced motor-evoked potentials (MEPs) in both forelimb and hindlimb muscles. Once the balloon reached 100 pounds per square inch (PSI) and during the subsequent 10 min, MEPs in hindlimb muscles were universally abolished in all animals, while spinally evoked potentials remained unchanged. This suggests that the injury caused damage to descending motor pathways without affecting the spinal reflex circuitry below the level of compression. The MEPs in hindlimb muscles recovered 8 days post-surgery (Figure 1).

### Neurofunctional testing

All animals exhibited normal gait function at baseline (BBB score 21). After injury, neurophysiological data was available for 3 of the 5 animals (due to 1 animal expiring and 1 animal not having undergone video recording). Of the 3 animals for which neurophysiological data was available, immediate post-injury BBB scores ranged from 4–17 (mean 9), with endpoint BBB scores ranging from 9–20 (mean 16.3). In animal #4, post-operative functional deficits were relatively mild, whereas in animals #3 and #5, post-operative deficits were more severe. Both animals #4 and #5 experienced considerable recovery of function by endpoint testing. Animal #3 experienced only modest recovery of function by endpoint testing, although this animal’s endpoint was relatively soon after injury (8 days) (Table 1).

**Table 1:** Neurofunctional outcomes.

Neurofunctional outcomes (BBB)			
Animal #	Preop	Postop	Final
1	ND (not recorded)	ND (not recorded)	ND (not recorded)
2	21	ND (expired)	ND (expired)
3	21	4 (POD 2)	9 (day 8)
4	21	17 (POD2)	20 (day 8)
5	21	6 (POD0)	20 (day 14)

Neurofunctional Scores (BBB) for 5 pigs undergoing experimental balloon-based thoracic spinal cord injury. Preop, pre-operative; Postop, post-operative.

### Histology

**Figure 2:**
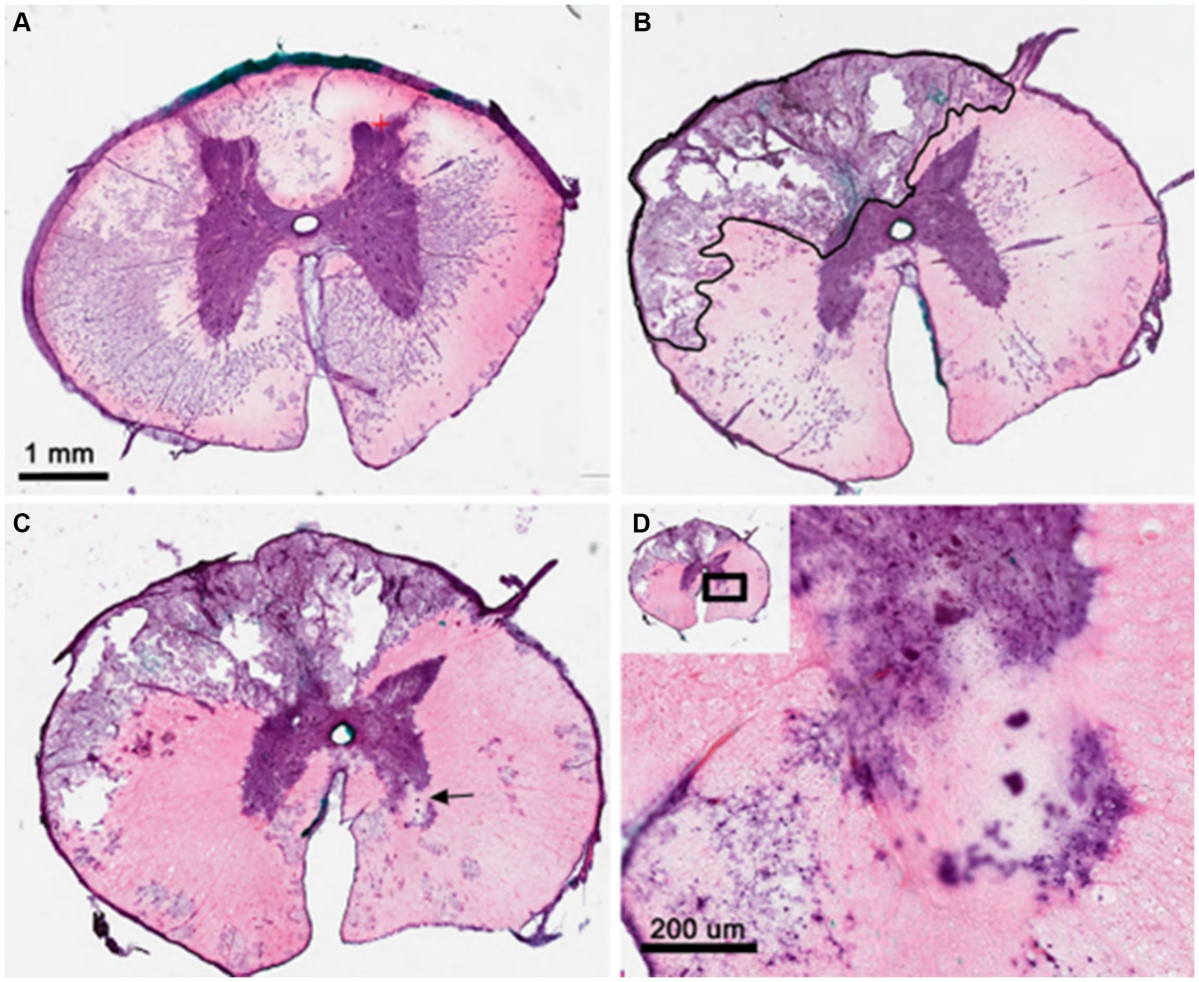
Histologic analysis of dorsolateral compression injury of the thoracic spinal cord from pig #4. **(A)** H&E staining of T10 spinal segment showing normal thoracic gray and white matter structures. **(B)** T12 segment at site of balloon compression shows cystic cavitation in the dorsolateral white matter and ipsilateral dorsal horn. Black outline indicates early cavitation perimeter with sparse axonal sparing and inflammation. **(C)** 100 mm caudal to lesion epicenter showing multi-lobular nature of cystic cavity and evidence of contralateral gray matter stroke (arrow). **(D)** High magnification of contralateral ventral horn showing evidence of both ventral horn motor loss and scarring as well as neighboring white matter damage. H&E, hematoxylin and eosin; mm, millimeters.

Post-mortem examination did not demonstrate any systemic (e.g., cardiopulmonary) pathologies in any animal. Endpoint spinal cord histology in each case demonstrated multilobular cystic cavitation at the site of injury, which was typically unilateral (Figures 2A–C). Sparse axonal sparing was seen within each cavitation perimeter along with signs of inflammation (Figures 2B,C). Significant axonal loss was observed in the dorsal and lateral funiculi producing damage to the anatomical location of descending motor corticospinal, rubrospinal and vestibulospinal tracts as well as ascending sensory tracts. Gray matter damage was limited to the ipsilateral dorsal horn and the superficial contralateral dorsal horn. In some cases, contralateral injury and/or ischemic infarcts were seen in the ventral horn with neuronal cell loss (Figure 2D).

## Discussion

The ideal animal model for SCI would closely and reproducibly mimic the scale and physiological consequences of SCI seen in humans. Large animal models hold significant advantages to model the scale and physiological consequences of spinal trauma but are limited in use due to expense, limited injury technology and the complexity of surgery and recovery. Here we have generated an innovative, minimally invasive spinal compression model in a large animal that is reproducible and mimics much of the pathophysiology of human spinal cord injury while maintaining the integrity of the spinal column and related structures. This model is well-suited for conducting quantitative assessments of sensorimotor physiology and imaging to evaluate lesion severity and is a potential tool for exploratory research and translational testing of surgical, neuromodulatory, and molecular repair approaches in the context of spinal injuries.

The first documented spinal cord injury animal model was a canine laminectomy and weight-drop model published by Alfred Allen in 1911 (21), and although advances in technology and understanding of spinal cord injury have led to improvements in this model, a large number of modern SCI animal models continue to utilize a contusion performed via an open surgical laminectomy (10). While contusion models do effectively reproduce a mechanism of SCI seen frequently in humans (22, 23), several limitations to the use of open laminectomy in animal models of SCI exist. Firstly, an open surgical laminectomy is an invasive procedure that requires muscle dissection and bony removal and leads to post-operative pain and morbidity. Laminectomy also carries risks such as durotomy, CSF leakage (1–15% in humans) (24), epidural hematoma (1% in humans) (25) and post-operative spinal instability (seen in 4.1–12% in humans) (12). Finally, laminectomy results in structural changes to the spinal canal which impact spinal cord perfusion pressure (14) and CSF flow dynamics (11) in ways that confound the investigation of spinal cord injury pathophysiology and therapeutics in an animal model.

### Minimally invasive models of SCI

Previously described minimally invasive animal models of spinal cord injury include models that minimize the amount of bone removed during the laminectomy portion of the injury surgery (15, 16), models involving microscopic dissection and/or operating techniques intended to minimize collateral tissue trauma (17), and models wherein a balloon compression device is inserted into the epidural or subdural space via a small hemilaminectomy defect (18–20). While each of these are less invasive than a typical open surgical laminectomy, they each still involve muscle dissection and bony removal, and are subject to the limitations of laminectomy in a spinal cord injury model discussed previously.

A canine SCI model described by Purdy et al. (26) utilizes an angioplasty balloon inserted percutaneously into the spinal subarachnoid space to induce a compression injury. This method foregoes the need for laminectomy or open surgery, and the histological and imaging results provided by the investigators are consistent with those seen in the currently described model but limited to 4 hours post-lesion. The authors utilized a balloon inflation pressure of 10 atmospheres (atm) in the majority of animals and left the balloon inflated for a period of 30 min. The authors’ description of the model is limited to the acute period and does not include neurofunctional assessments, electrophysiologic assessments, or myelin-based MRI imaging assessments, as were performed in our study. Dynamic, intraoperative electrophysiologic assessments were particularly important in our described model, as balloon inflation pressure was tailored to each animal (i.e., inflation was stopped at such time that transcranial MEPs were found to be absent in the lower extremities) in an attempt to create a reproducible, titratable injury and mitigate variance in canal and spinal cord size between animals. Additionally, the post-injury histology in our study frequently demonstrated cystic cavitation and loss of descending and ascending white matter tracts as well as limited motor neuron loss within the ventral horn ipsilateral to injury, which was not seen in the model described by Purdy et al.

### Applications for balloon-based SCI animal models

Animal models for SCI would ideally recapitulate forms of SCI seen in humans, and traumatic SCI in humans often involves high-speed ballistic injury with forces exceeding those attainable through the relatively slow inflation of a balloon, as was utilized in our study. Balloon inflation models may, however, appropriately reproduce the underlying mechanisms of spinal cord contusion seen in patients with gradually developing compressive pathologies such as epidural hematoma, epidural abscess, or degenerative stenosis. Additionally, pathologies such as cervical myelopathy (wherein spinal cord injury occurs due to very slowly progressive spinal cord compression) or spinal cord compression via a spinal tumor with epidural extension could theoretically be modeled with the use of slow and prolonged balloon expansion (27). The model developed here would also allow the redirection of the balloon to achieve either the midline, dorsolateral or even ventral compression.

### Limitations and future approaches

Our study possesses several limitations. Firstly, the sample size of the study was relatively small (5 animals). In addition, the method of neurofunctional testing used (BBB score via recorded video) is subject to bias. Furthermore, while the balloon inflation pressure utilized was governed by electrophysiologic loss of motor potentials in the lower extremities, the balloon inflation time utilized (15 min) was arbitrarily chosen, and balloon inflation was performed manually while a mechanical inflation may be more rigorous. Insertion of a kyphoplasty needle into the epidural space of a pig is technically challenging, as the Jamshidi needle used for insertion is of relatively large bore, and very little subarachnoid space is present in the thoracic spine of Yucatán pigs. The technique could possibly be improved upon with the use of CT-guided navigation, or experimentation with a transforaminal - rather than interlaminar - insertional approach. Moreover, the neurofunctional outcomes in the study were evaluated using the Basso, Beattie and Bresnahan (BBB) score, which is designed for use in rodents. While efforts have been made by other investigators to create a scale to characterize gait in pigs, no real consensus exists regarding gait outcomes scoring in pigs. Given this, the authors utilized the BBB score, which is widely utilized to evaluate gait function after spinal cord injury in rodents, but its relevance to gait function outcomes in pigs is unclear. Finally, the injury observed in the majority of animals was clinically, radiographically and histologically very mild, and as a result additional titration of injury severity may be necessary in order to achieve injury magnitudes bearing greater clinical relevance for therapeutic studies. Additional work is also necessary to better define differences in injury severity and morphology findings that will undoubtedly occur with variation in the time that the balloon is left inflated, the circumferential position of the catheter or variation in the speed of its inflation. The expense of performing these experiments may limit the utility of the model but the potential exists to produce distinct models that closely mimic a variety of common human SCI pathologies.

## Materials and methods

### Institutional approval

All animal experiments were performed with the approval of the institutional animal care and use committee (IACUC).

### Animals

**Figure 3:**
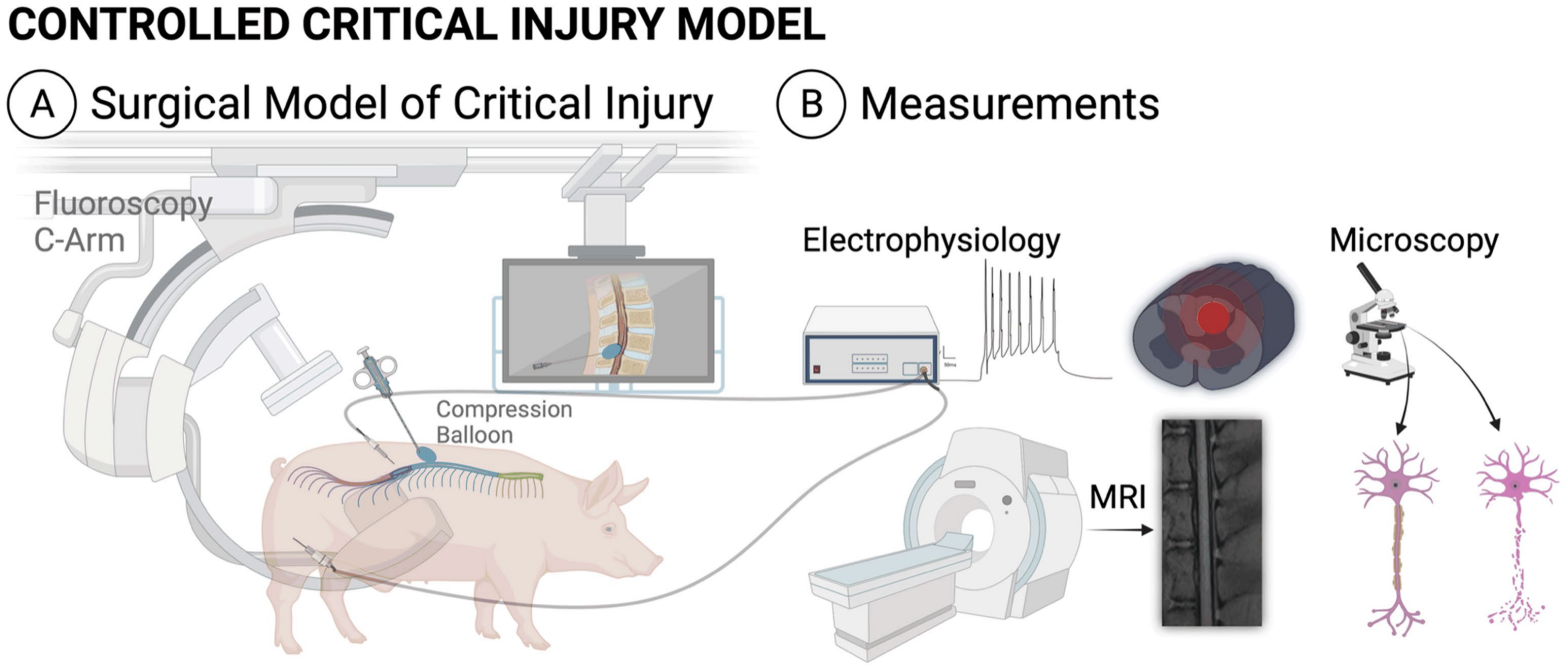
Illustration of the experimental protocol, including the surgical model **(A)** and experimental measurements **(B)**. All animals underwent pre-injury MRI, electrophysiologic testing and neurofunctional testing as well as immediate post-injury MRI and electrophysiologic testing. At endpoint, each animal underwent final neurofunctional testing, MRI and histology. MRI, magnetic resonance imaging.

Five healthy femaleYucatán pigs were utilized for the model. Mean pig age was 8 months at the time of surgery (range 3 months – 21 months). Mean weight was 44.4 kg (range 18–103 kg). Animal demographics are described in Table 2. Animals were sourced by the veterinary staff of our institutional Comparative Medicine Program. Prior to the injury surgery, all animals underwent baseline MRI, baseline neurofunctional testing and baseline neurophysiological testing. A diagram depicting the overall experimental protocol is seen in Figure 3. Post-operatively, animals were monitored by veterinary staff and received frequent direct observations and analgesia administration, as needed.

**Table 2:** Animal age, weight and other demographic characteristics.

Pig number	Strain	Age at surgery	Weight at surgery (kg)	Surgery to euthanasia timespan (days)
1	Yucatán	21 m 14d	103	5
2	Yucatán	3 m 21d	18	0
3	Yucatán	4 m 9d	25	8
4	Yucatán	6 m 5d	40	29
5	Yucatán	6 m 24d	35.8	27

### Injury surgery

**Figure 4:**
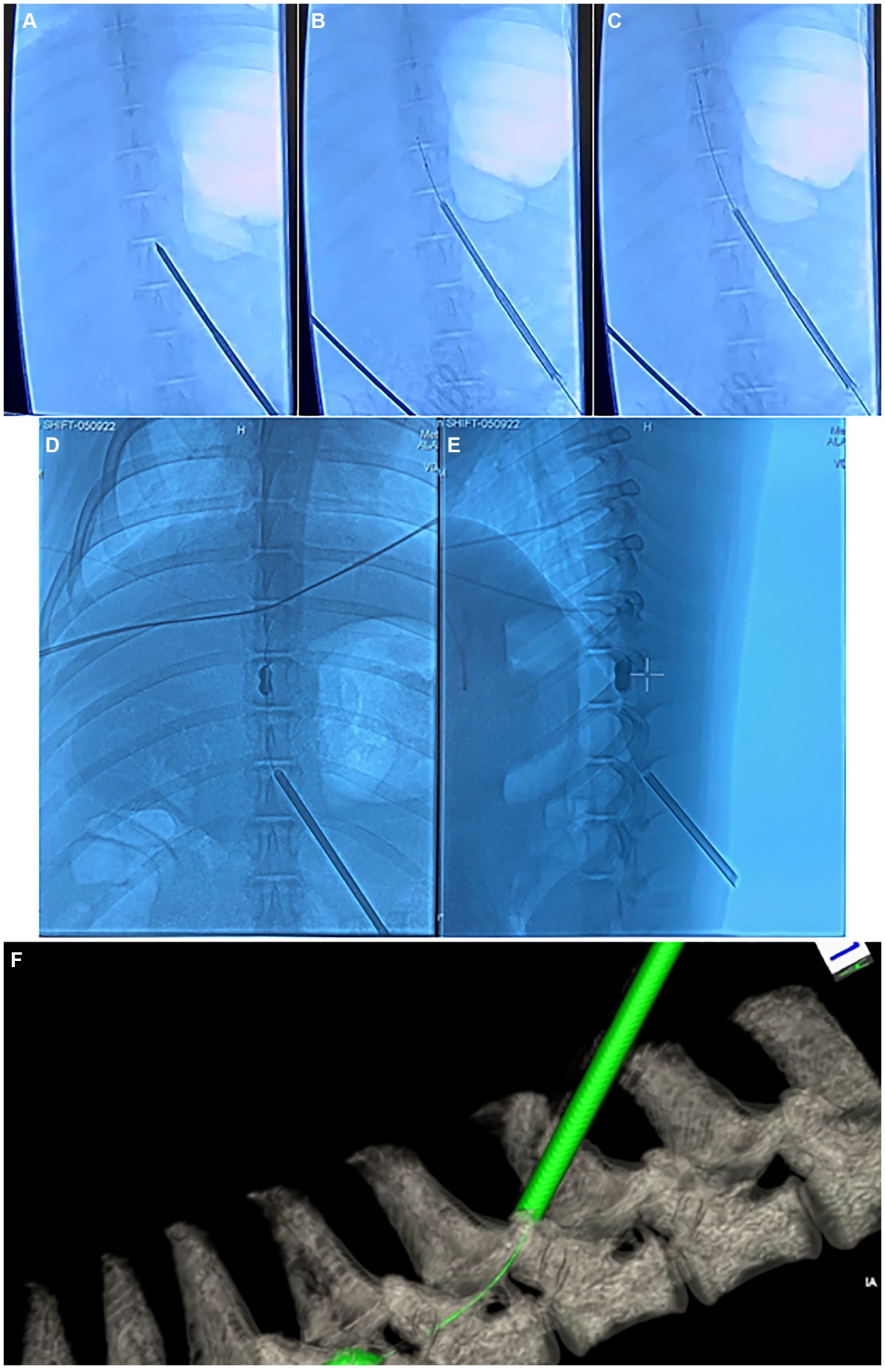
Fluoroscopy and computed tomography (CT) images depicting the injury surgery. **(A–C)** anterior–posterior (AP) fluoroscopy images depicting the insertion of the Jamshidi needle into the interlaminar epidural space in the thoracic spine, along with deployment of the kyphoplasty cannula into the epidural space. **(D)** AP and **(E)** lateral fluoroscopy images depicting inflation of the kyphoplasty balloon within the epidural space. **(F)** Dyna-CT depicting the kyphoplasty catheter and inflated balloon within the epidural space.

All animals received pre-operative penicillin and underwent general anesthesia via total intravenous technique (TIVA) using propofol in order to allow for testing of evoked potentials. Animals were placed prone on an operating table and were affixed to the table with straps. The T6 level was localized in each animal using fluoroscopy. A small incision was made in the left paramedian T10 area (4–6 cm lateral to the midline) and a Jamshidi needle was introduced into the T8/9 interlaminar space using fluoroscopy. From here, a kyphoplasty balloon (Kyphon, Medtronic, Inc., 8 gauge catheters, 15 mm diameter, 5 cc full inflation volume, 700 psi-rated) was introduced through the Jamshidi needle into the epidural space. The kyphoplasty balloon was advanced until the balloon was seen to be present laterally at the T6 level in order to create a lateralized injury. From here, the balloon was slowly inflated while running continuous motor-evoked potentials (MEPs). Once the MEPs in the lower extremities were seen to be absent, balloon inflation was stopped and the balloon was maintained at its current pressure for 15 min, after which time, the balloon was deflated and the balloon and Jamshidi needle were removed. Balloon inflation pressure amounted to roughly 15 psi in most cases, although inflation volume was not stopped according to balloon pressure, but rather according to the pressure at which MEPs in the lower extremities were abolished. The wound was closed in standard layered fashion. Final skin closure was with a topical skin adhesive. The animal was maintained under anesthesia for a post-injury MRI as well as post-injury neurophysiologic testing, after which time the animal was awoken from anesthesia. Fluoroscopy images depicting the kyphoplasty balloon deployment and inflation within the epidural space are seen in Figure 4.

### MRI

Each animal underwent baseline 3 T MRI on the day of injury, prior to the injury surgery, under general anesthesia. Traditional MRI sequences (T1, T2, STIR) images were acquired as well as myelin SHIFT MRI, which is a protocol intended to evaluate for the presence and character of myelin within the spinal cord and has been described in detail elsewhere (28). After the injury surgery, while still under general anesthesia, the animals underwent post-injury 1.5 T MRI, again with standard sequences as well as a myelin SHIFT protocol. Finally, at each animal’s endpoint, animals underwent final endpoint 3 T MRI under general anesthesia utilizing standard sequences and myelin SHIFT protocols.

### Electrophysiology

Baseline electrophysiology tests were performed for each animal on the day of injury, prior to injury, while the animal was under general anesthesia. Electrophysiology tests were also performed during the injury surgery, immediately after the injury surgery, and at animal endpoint.

*Electromyography (EMG)*: Subdermal 20 mm needle electrodes (Rhythmlink Columbia, SC, United States) were applied bilaterally to the left and right extensor carpi radialis (ECR), flexor carpi ulnaris (FCU), cranial tibial (CT), and gastrocnemius (GAS) muscles. A differential amplifier Octal Bio Amp (ADInstruments, Australia; gain: 100, range: ± 200 μV, resolution: 100 nV, common-mode rejection ratio: > 60 dB, input impedance: > 1 GOhm, < 100 pF) was used to amplify the recorded signals. Data were sampled at 10,000 Hz using a PowerLab data acquisition system (ADInstruments) and recorded using LabChart ADInstruments (version 8.1.24). The reference for the amplifier was a subdermal needle electrode positioned over the right calcaneal tuberosity.

*Transcutaneous spinal stimulation (TSS)* was delivered to the skin over the approximate location of the lumbosacral spinal enlargement (29) using an electronically controlled constant-current stimulator DS8R (Digitimer Ltd., United Kingdom). The physiologic state of motor pools and interneuronal networks in the lumbosacral enlargement was evaluated for each animal using this non-invasive approach, assessing the magnitude of spinally evoked motor potentials (SEMP) in the hindlimb muscles. Stimulation was administered using a self-adhesive cathode (diameter 5 cm; PALS, Axelgaard Manufacturing Co. Ltd., United States) placed along the midline of the spine between the L4 and L5 spinous processes. One oval anode (size 7.5 cm x 13 cm; PALS, Axelgaard Manufacturing Co. Ltd., United States) was placed on the abdomen. TSS was administered using single 500 μs monophasic square-wave pulses every 5 s. TSS started at 10 mA, increasing incrementally by 5 mA, reaching a maximum of 150 mA or until the magnitude of the somatosensory evoked potentials (SMEP) reached a plateau.

*Transcranial electrical stimulation* was administered through a constant-current stimulator (DS8R, Digitimer Ltd., United Kingdom). Two self-adhesive electrodes (diameter 3.2 cm; PALS, Axelgaard Manufacturing Co. Ltd., United States) were positioned along an imaginary line between the ears and approximately 3–4 cm toward the eyes, targeting the motor cortex (specifically referring to the C3/C4 position in the international 10–20 system of human EEG) (30, 31). Motor evoked potentials (MEPs) in the forelimb and hindlimb muscles were elicited using five pulses of 500 μs width, with interstimulus intervals of 1 ms (31), commencing at 30 mA and increasing in 10 mA increments, reaching up to 200 mA or until MEP magnitude plateaued. The supra-motor threshold MEP monitoring in the hindlimb muscles was conducted intraoperatively during the surgery as a means of determining the injury occurrence and severity through signal attenuation.

### Neurofunctional testing

Neurofunctional testing was performed for each animal immediately prior to the injury surgery, after recovery from the injury surgery (no greater than 1 week post-operatively), and once weekly after the injury surgery until animal endpoint. An “open field” behavioral observation method was utilized to gauge the animal’s neurological function. Each pig’s cadence up and down a secluded section of the animal vivarium hallway was recorded for 5–15 min each instance. Recording was performed via a wireless camcorder with a small tripod. Gait function was scored post-hoc via an independent observer utilizing the Basso, Beattie and Bresnahan (BBB) score (32).

### Endpoint and histology

At each animal endpoint, each animal underwent final neurofunctional assessment and was then once again placed under general anesthesia, after which time they underwent endpoint neurophysiological testing and endpoint MRI followed by euthanasia with barbiturate overdose. Transcardial perfusion was performed under general anesthesia using 4% paraformaldehyde. After euthanasia, tissues were harvested from each animal and histology was performed. Spinal cords were embedded in optimal-cutting-temperature (OCT) compound. A Microm HM450 microtome was used to cut spinal cord sections (20um each). Frozen sections were air dried overnight for staining.

Hematoxylin and eosin stains were performed as follows: Xylene bath for 1 min, Xylene bath for 2 min, additional Xylene bath for 2 min; 100% Ethanol bath for 30 s, second 100% Ethanol bath for 30 s; 95% Ethanol for 30 s; ddH2O bath for 30 s, Hematoxylin staining for 3 min, tap water rinse, acid alcohol (3 dips), tap water rinse, bluing Reagent for 1 min, tap water rinse, 95% Ethanol for 30 s, Eosin staining for 30 s, 95% Ethanol for 1 min, 100% Ethanol for 2 min, 100% Ethanol for an additional 2 min, Xylene for 2 min, Xylene for an additional 2 min, Xylene for a final 2 min, coverslip application.

#### Significance statement

Few large animal models exist that have the potential to reproduce the mechanisms of spinal cord injury (SCI) commonly seen in humans, which in turn limits the relevance and applicability of SCI translational research. SCI research relies heavily on animal models, which typically involve an open surgical, dorsal laminectomy which is inherently invasive and may have untoward consequences on animal morbidity and spinal physiology that limit translational impact. We developed a minimally invasive, large animal model of spinal cord injury which utilizes a kyphoplasty balloon inserted percutaneously into the spinal epidural space. Balloon inflation results in a targeted, compressive spinal cord injury with histological and electrophysiological features directly relevant to human spinal cord injury cases without the need for invasive surgery.

## Data availability statement

The original contributions presented in the study are included in the article/supplementary material, further inquiries can be directed to the corresponding author.

## Ethics statement

The animal study was approved by Houston Methodist Institutional Animal Care and Use Committee. The study was conducted in accordance with the local legislation and institutional requirements.

## Author contributions

SB: Conceptualization, Data curation, Formal analysis, Investigation, Methodology, Validation, Writing – original draft, Writing – review & editing. TW: Conceptualization, Data curation, Investigation, Methodology, Software, Writing – review & editing. AS: Data curation, Investigation, Methodology, Writing – review & editing. KH: Data curation, Investigation, Methodology, Writing – review & editing. MH: Data curation, Investigation, Methodology, Writing – review & editing. AF: Data curation, Investigation, Methodology, Writing – review & editing. XT: Data curation, Investigation, Methodology, Writing – review & editing. DS: Conceptualization, Data curation, Formal analysis, Investigation, Methodology, Supervision, Writing – review & editing. PH: Funding acquisition, Investigation, Supervision, Writing – review & editing.
